# Supplementary material for: Optimising Hollow-Structured Silicon Nanoparticles for Lithium-Ion Batteries
Source: Materials (Basel). 2023 Aug 28;16(17):5884. doi: 10.3390/ma16175884 (PMC10488427; doi:10.3390/ma16175884)
Supplement: Supplementary file 1 [file materials-16-05884-s001.zip › materials-2535213-supplementary.pdf]

Supporting Information

Optimising Hollow-Structured Silicon Nanoparticles for Lithium-Ion Batteries

X-ray Powder Diffraction

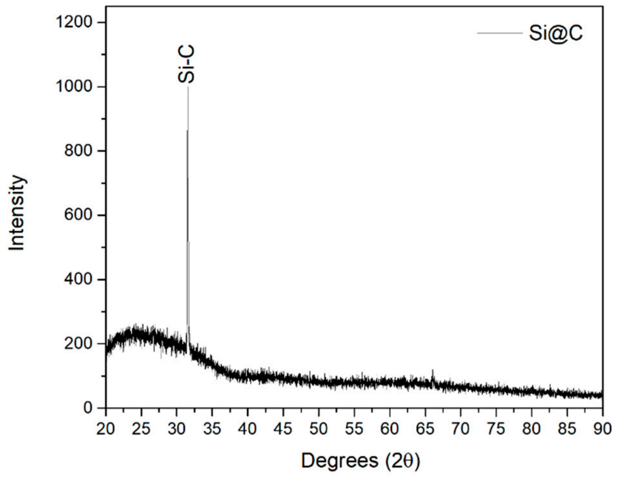

Figure S1. XRD spectrum obtained from Si@C.

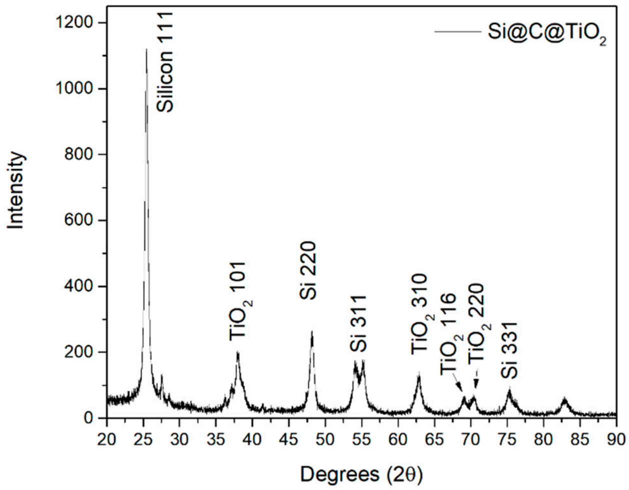

Figure S2. XRD spectrum obtained from Si@C@TiO2.

Half-cell and full-cell cycling

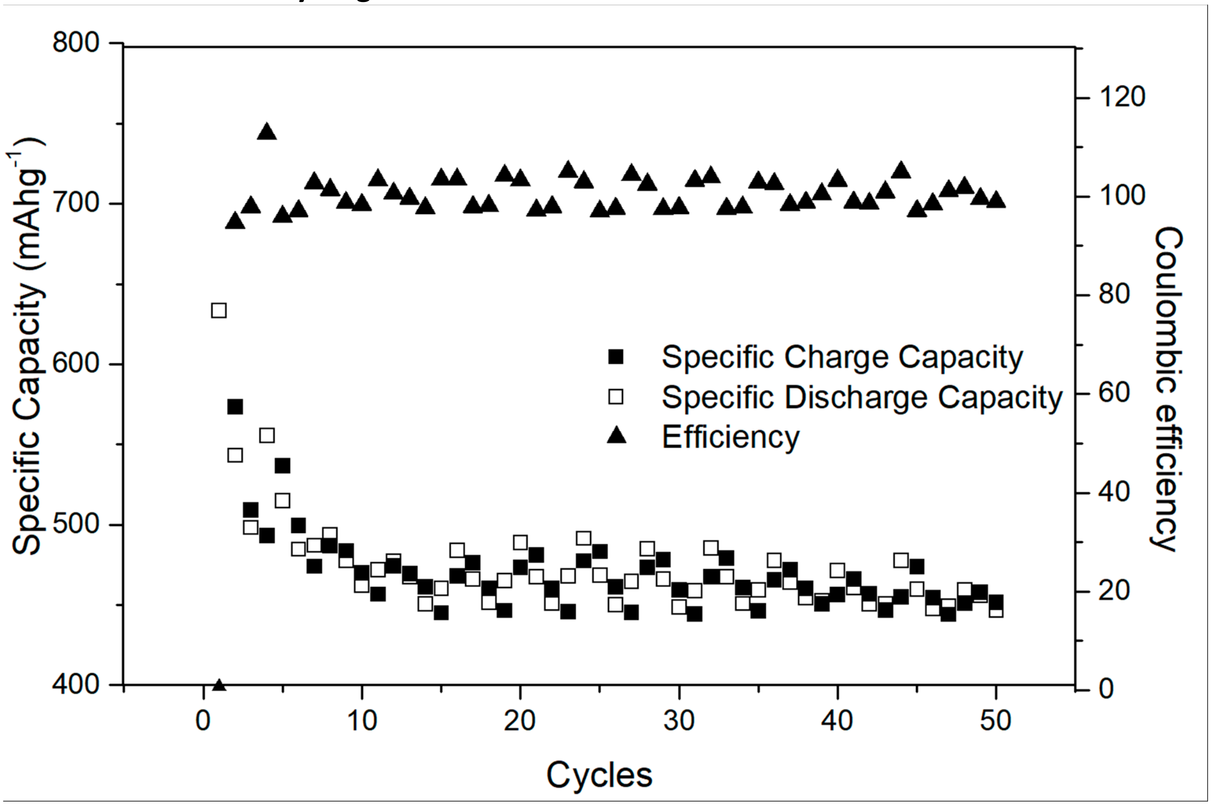

Figure S3. Half-cell specific capacity from Si@TiO$_2$ (low amount) and coulombic efficiency from Si@TiO$_2$.

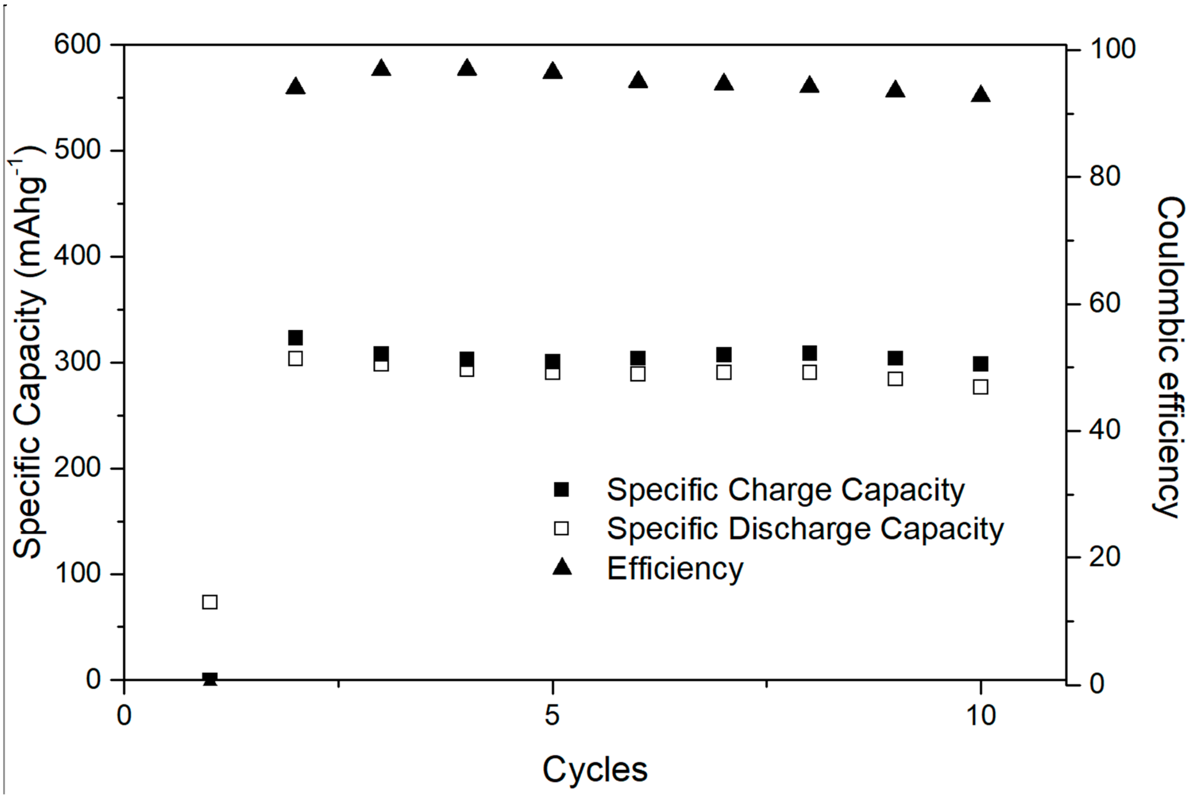

Figure S4. Full-cell specific capacity from Si@TiO$_2$ (low amount) and coulombic efficiency from Si@TiO$_2$.
